# Unmet supportive care need and associated factors among cancer patients

**DOI:** 10.1186/s12885-025-14607-w

**Published:** 2025-08-02

**Authors:** Woinab Degu Mehari, Yinghui Sun, Yunhong Lei, Sisay Shine, Elzabeth Seyoum, Mikiyas Amare Getu

**Affiliations:** 1 Department of Public Health, Yekatit 12 Hospital Medical Colleges, Addis Ababa, Ethiopia; 2 School of Nursing, Qilu Medical University, Shandong, China; 3 Academic Research Center, Yichang Hubo Medical Research Institute, Hubei, China; 4 Department of Reproductive and Family Health, School of Public Health, Addis Ababa University, Addis Ababa, Ethiopia; 5 College of Health Sciences, Woldia University, Woldia, Ethiopia

**Keywords:** Unmet needs, Supportive care, Cancer

## Abstract

**Introduction:**

Identifying and managing unmet supportive care needs is a vital aspect of providing comprehensive healthcare to cancer patients. Planning and redesigning services can be informed by assessment of unmet supportive care need.

**Objectives:**

This study aimed to assess unmet supportive care needs and associated factors among cancer patients in Ethiopia.

**Methods:**

A cross-sectional study design was conducted among 260 cancer patients. The sample size was obtained using a simple random sampling method. The collected data were entered and cleaned using EpiData 4.6 and exported to SPSS version 25 for analysis. Binary and multiple logistic regression analyses were performed to identify factors associated with the outcome variable. In the multivariate analysis, adjusted odds ratio (AOR) with 95% confidence interval (CI) were used as measures of association, and a *p*-value of less than 0.05 was considered statistically significant for unmet supportive care needs.

**Results:**

A total of 260 individuals were initially approached to participate, yielding a response rate of 93.1%. One hundred eleven participants (45.9%) reported at least one unmet supportive care needs. The most prevalent unmet supportive care needs were physical needs (61.6%) and health system needs (62.4%). Logistic regression analysis revealed age, sex, educational level, and stage of cancer diagnosis were significantly associated with unmet supportive care needs.

**Conclusion:**

The study highlights a high prevalence of unmet supportive care needs among cancer patients, primarily related to physical needs. The findings suggest potential opportunities for intervention in addressing physical needs. Age, sex, educational level, and stage of cancer at diagnosis were significantly associated with unmet supportive care need. Addressing these factors through tailored interventions can improve patient outcomes and guide policy decisions aimed at reducing unmet cancer support services.

## Introduction

Cancer is a leading cause of morbidity and mortality worldwide, with an increasing incidence driven by aging populations, changing lifestyles, and improved diagnostic capabilities [1]. Supportive care primary aims to maximize patients’ and their families’ comfort, function, and social support at all phases of the disease [2]. Supportive care in cancer is an essential component of comprehensive cancer care, encompassing physical, psychological, social, and spiritual support to improve the quality of life for patients and their families. This integrated approach reflects our local health context and is grounded in well-established frameworks. Fitch et al. (2008) [3] emphasize the multidimensional nature of supportive care by detailing key components such as psychosocial, physical, and informational support, as well as the importance of multidisciplinary collaboration in care delivery. More recently, Krishnasamy et al. (2023) [4], proposed an updated framework that integrates contemporary perspectives, emphasizing tailored interventions and patient-centered strategies across diverse healthcare settings. Supportive care is a multidisciplinary strategy that neither hastens nor delays death, but if it is initiated early, it can have a favorable impact on the course of the illness.

Unmet supportive care needs can lead to increased patient distress, poor adherence to treatment, and overall decreased quality of life. Early supportive care not only enhances patients’ quality of life but also lowers unnecessary hospitalizations and healthcare utilization [5]. For example, research has shown that patients who receive early supportive care experience a decreased symptom burden, thereby achieving fewer emergency department visits and lower rates of unplanned hospital admissions [5]. A wide range of illnesses require supportive care, and among adults who need it, cancer ranks second most frequently among chronic diseases (it affects 34% of adults, after cardiovascular disorders, which affect 38.5% [6].

One of the most frequent and important symptoms encountered by patients in need of supportive care is pain, which affects 80% of cancer patients at the end of their lives [7]. According to the WHO global action plan for the prevention and control of non-communicable diseases the world’s aging population, the growing burden of modifiable risk factors for cancer like smoking, obesity, physical inactivity, and adoption of a western lifestyle, globalization, urbanization, and economic development are all contributing to the rapid rise in the need for supportive care among cancer patients worldwide [8]. However, throughout the majority of the world, and particularly in sub-Saharan African nations, there is still a considerable unmet demand for supportive care for individuals with chronic, life-limiting health disorders, with a significant variation for cancer care [9].

In Ethiopia, cancer is an emerging public health challenge, exacerbated by the nation’s large population of approximately 110 million people, making it the second most populous country in Africa. Due to the alarming global population growth and changing lifestyles, the risk of cancer is expected to rise. However, the country is ill-equipped to handle this increasing burden, with only one fully operational public radiation center and a limited number of physicians and nurses available to provide oncology services nationwide [10]. In addtion to structural limitations, cancer treatment faces significant challenges, including continually rising cost of chemotherapy, limited availability of treatment options, drug resistance of cancer cells, and complexity and high cost of radiotherapy facilities and equipment.These issues are further exacerbated by inadequate supportive care services, which are essential for managing pain, alleviating symptoms, and improving the quality of life for cancer patients [11].

Cancer diagnosis and treatment result in significant physical and psychological distress in cancer patients, necessitating supportive care [12]. Supportive care in cancer plays a crucial role in managing the disease and its psychological and physical effects [13]. Evidence has revealed considerable unmet needs in five primary areas: patient care and support, physical and daily living, psychological support, health system and information, and sexual needs are the most important supportive care need components to improve patient health outcomes [14]. Several socioeconomic and clinical factors hinder the access to high-quality supportive cancer care in Ethiopia. These factors includes older age, male gender, longer time since diagnosis, and being unmarried, as well as clinical factors such as an advanced stage of cancer at diagnosis, a history of recurrence, the type of treatment, residence, and coexisting illnesses [15, 16]. Patients who use positive coping strategies exhibit better adjustment to cancer, experience fewer mental health problems, and face less psychological distress [17]. Additionally, support from government initiatives and healthcare organizations is instrumental in allocating resources and implementing programs that promote early intervention.

Understanding the magnitude of unmet supportive care needs and its associated factors contribute to inform policy and healthcare planning, ensuring that resources are allocated effectively to improve patient outcomes. This research also addresses the disparities in access to care, particularly for marginalized populations, and drive efforts to establish more equitable healthcare services. The finding of this study will also guide the development of targeted interventions to enhance the well-being of cancer patients.

Therefore, this study aimed to assess the magnitude of unmet supportive care needs and associated factors among cancer patients in Ethiopia.

## Methods

### Design and sample

A cross-sectional study was conducted at Tikur Anbessa Specialized Hospital (TASH), the largest tertiary hospital in Ethiopia, where patients are referred from all parts of the country. The study was carried out from January 1 to April 30, 2023. The inclusion criteria included: (1) cancer patients undergoing treatment during the study period; (2) aged 18 years or older; and (3) having received therapy at least once before. Patients who were severely ill and unable to communicate during the data collection period were excluded. A patient was considered severely ill if they were experiencing acute complications or clinical condition that impaired their ability to engage in the data collection process, such as being in advanced stage of disease with life threatening conditions. Unable to communicate was defined as patients who were not able to respond basic questions due to cognitive impairment, language barriers or other conditions.

The sample size was determined using a single population proportion formula, with an unmet supportive care need proportion of 81% based on a prior study conducted at Dessie Referral Hospital [18]. This estimate reflects the proportion of cancer patients who reported unmet supportive care needs in that setting. A 95% confidence level and a 0.05 margin of error were applied. By considering for a 10% non-response rate, the final sample size was 260. A systematic random sampling technique was used to select study participants.

### Data collection instruments

#### Supportive Care Needs-Short Form (SCNS-SF25)

The data was collected by a validated and psychometrically tested questionnaire called SCNS-SF25. The instrument was designed to assess patents needs across five domains: psychological, health system and information, physical and daily living, patient care and support and sexuality needs [19].

A 5-point Likert scale was employed, where a rating of 1 indicated (not applicable/not a problem), 2 indicated satisfied, and ratings of 3, 4, and 5 represented low, moderate, and high levels of unmet need, respectively. Values of 1 and 2 were rated as (No Need) and 3, 4 and 5 as (Some Need). For analysis, the scores were treated as categorical measures, where a rating of 3 or higher was considered to indicate an unmet need. Additionally, subscale scores were calculated by summing the individual items. A higher score indicated a higher perceived need. Alternatively, the scale can provide information on the presence and number of perceived unmet needs, with a rating of 3 or higher considered as unmet need. Participants were instructed to circle the number that best described whether they needed help with each statement in the past month. The validity and reliability of the Amharic version of the SCNS-SF25 have been established in a study conducted at Hawassa referral hospital consisting of 25 items with overall Cronbach’s alpha of 0.933, ranging from 0.755 to 0.994 for the five domains [15].

#### Socio-demographic and clinical characteristics

The participants were asked about socio-demographic (e.g., age, gender, education, marital status, income, place of residency) and clinical information (e.g., age at diagnosis, date of diagnosis, cancer type, stage at diagnosis, and other comorbidities).

### Data quality assurance

Training was provided to data collectors and supervisors before the actual data collection. A pretest of questionnaire was conducted on cancer patients in another hospital, involving 5% of the sample size, prior to the main data collection. All complete data were examined for completeness and consistency during data management, storage, cleaning, and analysis. Additionally, the statistician entered and cleaned the data before analysis.

### Statistical analysis

The data was sorted, coded, and entered into Epi-Data 4.6, then exported to SPSS software version 25.0 for analysis. Descriptive statistics were performed to describe each variable using frequency, mean, and standard deviation. Binary and multiple logistic regression analyses were used to assess the relationship between each independent variable and the outcome variable. During binary logistic regression analysis, independent variables with a p-value less than 0.25 were included in a multivariable analysis to identify the significant factors associated with the outcome variable. A backward stepwise selection method was used to remove non-significant variables from the models. Adjusted odds ratios with 95% confidence intervals were used to assess the association between predictor variables and the outcome variable. The Hosmer-Lemeshow goodness-of-fit test was used to check model fitness. The level of statistical significance was set at a p-value of less than 0.05. Finally, the results were presented in frequency distribution tables, charts, and graphs.

## Result

### Patient characteristics

In this study, a total of 260 participants were initially approached and only 242 participants completed the questionnaires and giving a response rate of 93.1%. Among 18 non-respondents, systematic defferences were examined to assess potential biases in the data. These non-respondents were primarly those who were unable to participate due to severe illness, time constraints, or being absent during data collection period. An analysis of age, sex, cancer type, and treatment status revealed no significant difference between respondents and non-respondents. The mean age of the participants was 43.2 (SD ± 7.43) years. The majority of the participants were female (74.0%) and married (73.1%). More than three-fourth (89.7%) of participants completed pre-high school level of education. Nearly two-thirds (82.6%) of the participants were employed at the time of the study. The most common type of cancer was breast, accounting for nearly one-third (35.9%) of the participants. Colorectal cancer/gastrointestinal cancer is the second most common (24.4%), followed by hematological malignancies (20.0%). The majority of participants (86.4%) reported a cancer stage below stage IV. (Table 1)

**Table 1:** Socio-demographic and clinical characteristics of study participants (*n* = 242)

Characteristics		*N*	%
Age (years)	18–45	155	64
	46–60	59	24.4
	> 60	28	11.6
Gender	Male	63	26.0
	Female	179	74.0
Monthly income (ETB)	≤ 1500	119	49.2
	1500–2000	79	32.6
	2001–2700	25	10.3
	≥ 2700	19	7.9
Marital status	Single	65	26.9
	Married	177	73.1
Occupational	Employed	200	82.6
Status	Non-employed	42	17.4
Educational Status	Unable to write and read	56	23.1
	Primary education	57	23.6
	Secondary education	104	43.0
	Diploma and above	25	10.3
Residence	Rural	120	49.6
	Urban	122	50.4
Primary cancer site	Breast	87	35.9
	Hematological malignancies	48	20.0
	Colorectal/gastrointestinal	59	24.4
	Lung	15	6.2
	Prostate	21	8.7
	Skin	12	4.9
Treatment received in the last month	Chemotherapy	181	74.8
	Radiotherapy	61	25.2
Disease Recurrence	Yes	223	92.1
	No	19	7.9
Comorbidity	Yes	58	24.0
	No	184	76.0
Stage of tumor	I	33	13.6
	II	140	57.9
	III	36	14.9
	IV	33	13.6
Treatment side effect	Yes	225	93.0
	No	17	7.0

*ETB* Ethiopian Birr

### Information status about patient diagnosis

Out of the total patients, 209 (86.4%) received information about their diagnosis. One fourth (25.6%) were provided information about the possible cause of their disease. Additionally, nearly all 222 (91.7%) were informed about the medical evaluation for diagnosis of their disease. Regarding medical evaluation, 61 (25.2%) of participants were informed about the evaluations themselves, while 223 (92.1%) of participants received information about their medical treatment. In terms of treatment sequences, 182 (75.2%) had information about the order of treatments they received. Sixty-two (25.2%), eighteen (33.1%) and one hundred eight (44.6%) were informed about the expected benefits, duration, and possible side effects of their treatment respectively. Among the patients, 205 (84.7%) received information from health professionals, while the remaining participants obtained information through other patients 22 (9.1%) and family members 15 (6.2%). (Table 2)

**Table 2:** Participants information status about diagnosis of cancer (*n* = 242)

Variable	Response	*N*	%
The diagnosis of your disease	Yes	209	86.4
	No	33	13.6
The possible causes of your disease	Yes	62	25.6
	No	180	74.4
The purpose of any medical tests you have had	Yes	222	91.7
	No	20	8.3
The procedures of the medical tests	Yes	61	25.2
	No	181	74.8
The medical treatment (chemotherapy, radiotherapy, surgery or other treatment modality)	Yes	223	92.1
	No	19	7.9
The sequence of the medical treatments	Yes	182	75.2
	No	60	24.8
The expected benefit of the treatment	Yes	62	25.6
	No	180	74.4
The possible side-effects of your treatment	Yes	80	33.1
	No	162	66.9
Information about the duration of your treatments	Yes	108	44.6
	No	134	55.4
Source of information about diagnosis	Health professional	205	84.7
	Family member	15	6.2
	Other patients	22	9.1

### Prevalence of unmet supportive care needs

**Fig. 1:**
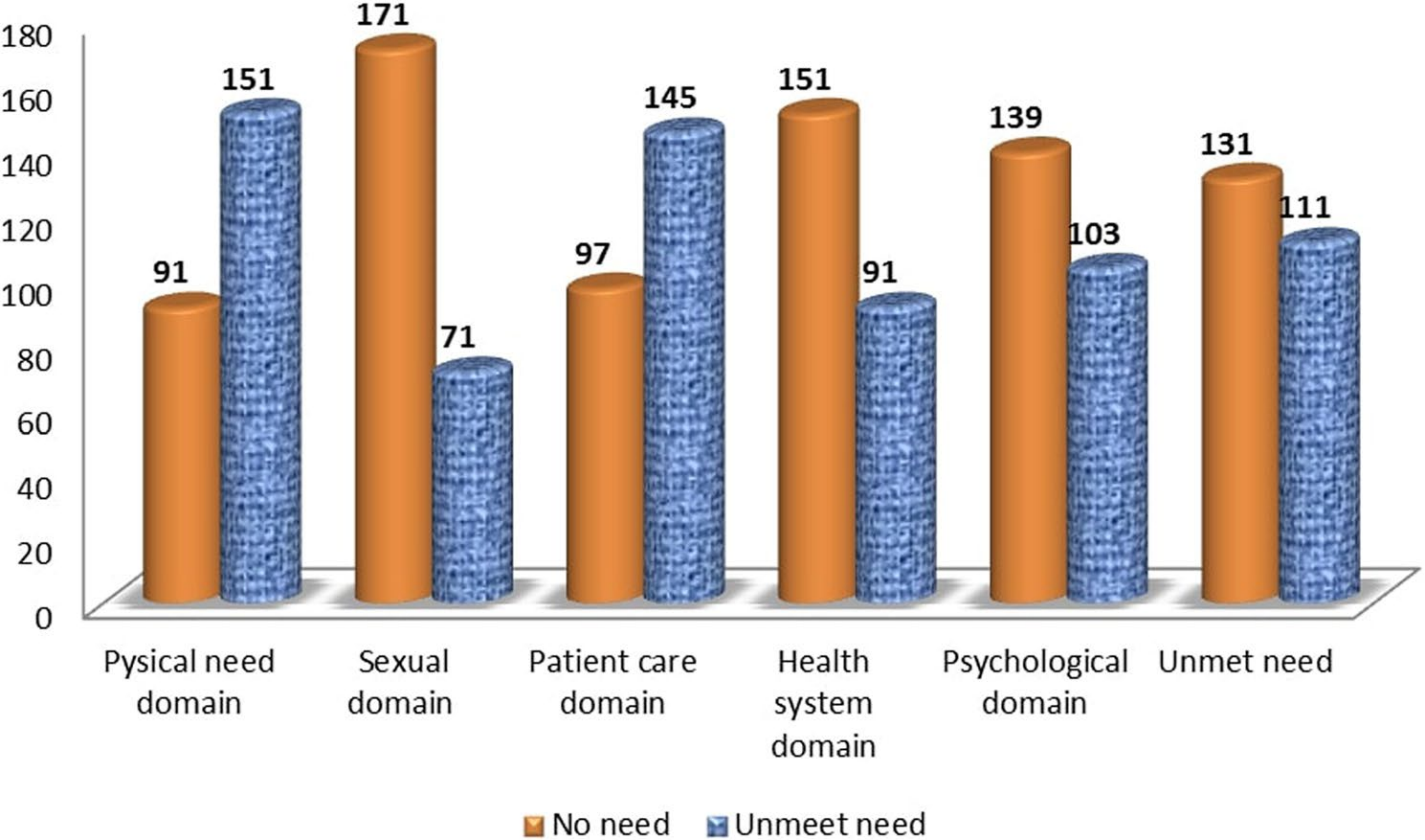
Prevalence of unmet supportive care needs among cancer patients in Ethiopia

Among all the participants, a total of 111 individuals (45.9%) were categorized as having unmet supportive care needs. Figure 1 provides a visual summary of the distribution of unmet needs and the categorization of supportive care among the participants in the assessment. The assessment revealed that the greatest needs were identified in the physical need domain 151(62%), patient care domain 145 (60%), followed by psychological domain 103(43%), health system domain 91 (38%), and sexual domain 71 (29%).

In the Psychological domain, the most prominent sub-scale was concern about the worries of others (79.8%). Within the Health system/information domain, the highest priority was receiving prompt notification of test results (86.4%). Feeling unwell most of the time dominated the Physical need domain (80.6%). In the Patient care/supportive need domain, the top concern was hospital staff promptly attending to physical needs (64.9%). Lastly, the Sexual domain highlighted a significant concern with changes in sexual feelings (84.3%). (Table 3)

**Table 3:** Unmet supportive care need among cancer patients in Ethiopia (*n* = 242)

Domain	Unmet supportive care need	*N* (%)
Psychological domain	Anxiety	124 (51.2)
	Feeling down or depression	108 (44.8)
	Feeling sadness	121 (50)
	Fearing about spread of cancer	183 (75.6)
	Worry about the treatment beyond control	118 (48.8)
	Uncertainty about the future	123 (50.8)
	Learning to feel in control of your situation	108 (44.6)
	Keeping a Positive outlook	84 (34.7)
	Feeling about death and dying	90 (37.2)
	Concern about the worries of those you	193 (79.8)
Health system/information domain	To be given explanations of those tests for which you would like explanations	179 (79.3)
	To be adequately informed about the benefits and side effects of treatments before you choose to have them	181 (74.8)
	To be informed about your test results as soon as possible	209 (86.4)
	To be satisfied with over all service	195 (80.6)
Physical need domain	Pain	189 (78.1)
	Lack of energy/tiredness	108 (44.6)
	Feeling unwell most of the time	195 (80.6)
	Work around the home	168 (69.4)
	Not being able to do things you used to do	194 (80.2)
Patient care/supportive care need domain	Reassurance by medical staff that you feel normal	229 (29.8)
	Hospital staff attending promptly to your physical need	157 (64.9)
	Hospital staff acknowledging and showing sensitivity to your feelings and emotional need	146 (60.3)
Sexual need domain	Change in sexual relationships	193 (79.8)
	Change in sexual feeling	204 (84.3)
	To be given information about	195 (80.6)

### Factors associated with unmet supportive care need

Bi-variate logistic regression was done for each independent variable, and eight variables were identified at p-value less than 0.25 to be fitted for multi-variable logistics regression. These variables were age, sex, educational status, monthly income, and disease remission, stage of cancer at diagnosis, comorbidity and treatment side effect.

In multi-variable logistic regression analysis age, sex, educational level, and stage of cancer at diagnosis were significantly associated with supportive care need among cancer patients. (Table 4) The finding of this study indicated that patients aged ≦ 45 had significantly higher odds of having unmet supportive care needs compared to those aged > 60 (AOR: 3.9, 95% CI: 1.81–13.89). Similarly, patients in the age group of more than 46–60 were 9.3 times more likely to have unmet supportive care needs compared to those aged more than 60 (AOR: 9.3, 95% CI: 1.29–16.52). This study also revealed that male patients had higher unmet supportive care needs compared to female patients (AOR: 4.3, 95% CI: 2.48–15.11).

**Table 4:** Factors associated with supportive care need in Ethiopia (*n* = 242)

Variables	Options	Supportive care				COR (95% Cl)	AOR (95% Cl)
		No need		Unmet need			
		*N*	%	*N*	%		
Sex of respondent	Female	42	20.2	137	5.8	1	1
	Male	49	17.3	14	56.6	11.4 (5.74–22.69)	4.3 (2.48–15.11)*
Age of respondent	≦ 45	58	24	107	44.2	0.10 (0.030–0.390)	3.9 (1.81–13.89)**
	46–60	18	7.4	41	16.9	1.23 (0.651–2.341)	9.3 (1.29–16.52)**
	> 60	15	6.2	3	1.2	1	1
Educational status	Unable to write and read	19	7.8	37	15.3	0.79 (0.310–2.029)	3.4 (0.69–16.77)
	Primary education	11	4.5	17	7	0.57 (0.294–1.131)	12.9 (1.48–19.46)**
	Secondary education	49	20.2	55	22.7	1.79 (0.770–4.193)	3.4 (1.76–15.20)
	Diploma and above	18	7.4	17	7.02	1	1
Monthly income	Less than equal to 1500	18	7.4	101	41.7	0.07 (0.031 - 0.164)	0.5 (0.05–0.11)
	1500–2000	62	25.6	17	7.4	1.45 (0.521–4.051)	1.9 (0.25–4.74)
	2001–2700	7	2.9	18	16	1.28 (0.504–3.242)	0.5 (0.13–2.07)
	Greater than 2700	4	1.7	15	6.2	1	1
Remission	Yes	70	28.9	117	48	0.97 (0.521 −1.80)	0.93 (0.35–2.46
	No	21	8.6	34	14	1	1
Stage of cancer at diagnosis	Early	42	17.3	27	11.2	1	1
	Late	129	53.3	44	18.2	1.88 (1.04–3.41)	1.97 (1.05–3.69)*
Comorbidity	Yes	22	9	36	14.8	1.9 (0.554–1.872)	2.48 (0.94–6.54)
	No	69	28.5	115	47.5	1	1
Treatment side effect	Yes	81	33.4	144	59	0.84 (0.45–1.58)	0.89 (0.28–3.89)
	No	10	4.1	7	2.9	1	1

*AOR* Adjusted Odds ratio, *CI* Confidence interval 1: Reference category; * *p* < 0.05, **p < 0.01

Regarding educational status, patients with primary education had a significantly higher odds of having unmet supportive care needs compared to those who are unable to read and write (AOR: 12.9, 95% CI: 1.48–19.46). After accounting for other potential confounding factors, cancer patients with a primary education level were found to be 12.9 times more likely to experience unmet supportive care needs compared to those with a diploma or higher education level. Additionally, patients with colorectal cancer demonstrated a significantly higher likelihood of reporting unmet supportive care needs compared to those with hematological malignancies (AOR = 7.28, 95% CI: 1.96–27.07). Although breast cancer patients also reported a higher proportion of unmet needs (41%), the association was not statistically significant (AOR = 1.08, 95% CI: 0.41–2.86).

Furthermore, the findings of this study showed that patients diagnosed with late-stage cancer had markedly greater chances of experiencing unmet supportive care needs in comparison to those diagnosed with early-stage cancer (AOR: 1.97, 95% CI: 1.05–3.69).

## Discussion

This hospital based cross-sectional study aimed to assess unmet supportive care needs and its associated factors among cancer patients. The overall unmet supportive care need score was found to be 45.9%, which is lower than other studies. For example, a study among cervical cancer patients in Nepal found that unmet needs in the psychological domain reached 87.9% [20], while studies in Japan and Korea reported overall unmet need rates of 76.7% and 91.8%, respectively [21, 22], and a German study documented a 72.1% prevalence across all domains [23]. These discrepancies may be attributed to differences in cultural perceptions of supportive care, variations in specific types and stages of cancer included in the studies. Additionally, differences in the measurement tools and thresholds for defining unmet needs could also contribute to these variations.

This study revealed that the most prevalent domains of unmet supportive care needs were physical needs and health system needs. These two domains accounted for a substantial majority, specifically 62%, of the overall unmet needs. This indicates that a significant proportion of the study participants experienced unresolved physical and health system issues in terms of supportive care. This was followed by unmet needs of patient care, sexuality and psychological domains. This finding is consistent with studies conducted in Singapore, Hong Kong, and Korea, where the health system domain ranked the highest mean score [24]. However, a research based in Australia [25] and Malaysia [26] demonstrated that cancer survivors identified psychological domain as having the highest level unmet supportive care needs. This finding is consistent with studies conducted in Nigeria, the United Kingdom, Australia, and the United Arab Emirates [27]. Additionally, a study in Northwest Ethiopia found that the psychological domain had the highest supportive care need [28]. One potential explanation for this variation could be that cancer survivors in urban areas tend to have greater awareness and access to various sources of information. This increased awareness may empower them to actively seek information to improve their health and prevent psychological distress resulting from a lack of understanding about the disease and its treatments.

This study also found that the sexuality domain had the second-lowest score after the psychological domain. This result is line with the studies conducted in Hong Kong and Chinese [29, 30], Iran [31], Nigeria [32], and Malaysia [26]. However, a study in the United States reported contrasting results, indicating that the sexuality domain had the highest level of supportive care needs among patients [33]. This difference could be attributed to cultural, religious, and ethical variations among these countries. In Ethiopia, the majority of patients adhere to culturally conservative norms and may be reluctant to disclose information regarding their sexual behavior.

The logistic regression analysis revealed that sex, education level, age, and cancer stage at diagnosis were significantly associated with supportive care needs. Notably, patients with a secondary education had significantly higher odds of experiencing unmet supportive care needs. This finding is aligned with previous study conducted in Korea, which also suggested that patients with lower levels of education may have limited resources to meet their needs compared to those with higher education [34]. The limited access to information about their illness and treatment among patients with low education level may contribute to their heightened unknown fears in comparison to individuals with higher education. Additionally, lower level of education might be associated with poorer health literacy, which can hinder patients’ understanding of available care opitions and their ability to advocate for their needs.

Our study found that sex as a significant predictor for supportive care needs, consistent with previous research conducted in Northwest Ethiopia [28] and other systematic review and meta-analyses in Sub-Saharan Africa [35], which found that females exhibited higher unmet needs compared to male patients. This underscore the influence of gender in determining the extent of supportive care needs among cancer patients, specifically in the patient care domain [28]. This gender disparity may be attributed to factors such as women being more proactive in seeking healthcare services and reporting their care needs, a trend supported by studies showing that female patients report higher levels of supportive care needs [36] and consistently prioritize care aspects more than men [37]. Traditional gender roles and expectations, prevalent in certain cultures, can influence how men and women express or seek help for their emotional and physical needs. Furthermore, the predominance of breast cancer in our study sample may also influence these findings, as patients with breast cancer are often more engaged in supportive care services.

Additionally, our study revealed a strong association between younger age [below 46 years) and unmet supportive care needs. This finding aligns with research conducted in Switzerland and Mexico, which also observed higher levels of unmet physical needs among younger individuals [38, 39]. Conversely, a study in Korea reported that younger age [below 50 years) was significantly linked to unmet needs in the sexuality and health system and information domains [40, 41]. This discrepancy may be attributed to the increased stress and disruption that cancer causes on younger patients, who may have poor coping mechanisms. These individuals often face additional challenges in adapting to the emotional, psychological, and physical toll of cancer. Furthermore, cultural norms in our study setting may inhibit open discussion of sexuality needs, potentially exacerbating unmet supportive care needs. In many cultures including Arabs, Chinese and in our study setting, discussions about sexuality and sexual health are often considered taboo or are subject to societal stigmas, which may prevent patients from seeking or receiving the necessary support [42–44]. This reluctance to address sexuality may lead to underreporting of needs and hinder healthcare providers from offering relevant care.

Monthly income emerged as a statistically significant predictor of supportive care needs among our study participants, in line with research from Korea, which concluded that low income predicted higher needs. Additionally, another study in Korea indicated that low-income patients were more likely to report unmet needs in physical and daily living domains [34]. This association suggests that patients with lower incomes may face challenges in meeting their daily personal care and physical needs. However, contrary to these findings, a study conducted in Northwest Ethiopia found no significant association between monthly income level and supportive care needs [28]. Similarly, an international unmet supportive care needs study reported low- and middle-income countries had significantly higher number of unmet needs compared to high income countries (HICs). In LMICs patients may face more barriers to accessing adequate care, contributing to higher levels of unmet needs, especially in domains related to physical and daily living requirements [45].

Furthermore, patients with late-stage cancer exhibited a greater need for supportive care compared to those with early-stage diagnoses. This aligns with findings from studies conducted in Ethiopia and Malaysia, where cancer survivors with advanced stage diagnoses reported greater physical and psychological needs [18, 26]. This likely reflects the increased complexity of care and the more intensive supportive care needs of patients with advanced cancer, which may not be adequately addressed in resource-limited settings. In contrast, a cross-sectional qualitative study in the Middle East and Taiwan found that patients with stage II, III, and IV cancer had significantly lower needs compared to those with stage I diagnoses. Another study reported disease stage had no siginificant impact on the level of supportive care need [46].

These discrepancies may be attributed to variations in study design, resulting in divergent outcomes across different study populations [47, 48].

### Strength and limitation of the study

The study’s comprehensive assessment of unmet supportive care needs among cancer patients, covering various domains such as physical, psychological, patient care, sexuality, and health system needs, stands as a notable strength. This holistic approach provides a thorough understanding of the challenges faced by cancer patients in accessing supportive care services. Additionally, the finding of the study could provide an evidence-based basis for healthcare decision-making and health service practice. Despite these strengths, the study is not without limitations. Cross-sectional study design restricts the ability to establish causality between variables, suggesting the potential for longitudinal studies to offer deeper insights into the dynamics of supportive care needs over time and the impact of interventions. Relying on self-reported data from cancer patients introduces the possibility of recall bias or social desirability bias, potentially affecting the accuracy of the findings.

### Clinical implication and future research

The study highlights the importance of developing tailored supportive care services that address the specific needs of cancer patients, particularly in domains such as physical care and the healthcare system. Ethiopian healthcare providers can use this information to prioritize interventions and allocate resources effectively, aiming to enhance patient outcomes. Additionally, recognizing gender disparities in supportive care needs, healthcare providers should adopt a gender-sensitive approach in delivering care and addressing the unique needs of male and female cancer patients. For example, women may benefit from additional psychological and emotional support services, while men might require more attention to physical care needs. In the context of Ethiopia’s healthcare system, where resources are often limited, it is useful to develop cost-effective and scalable interventions that can be integrated into existing healthcare structures. Healthcare providers should be trained to recognize and address specific domains of unmet needs, strengthening support systems for cancer patients, and improving access to services like pain management and psychosocial support. Looking ahead, longitudinal studies are needed to explore the trajectory of supportive care needs among cancer patients over time. Investigating the effectiveness of targeted interventions through intervention studies holds promise in identifying strategies to reduce unmet supportive care needs and improve quality of life [49]. Furthermore, supplementing quantitative findings with qualitative research methods could offer deeper insights into the subjective experiences and perspectives of cancer patients regarding their supportive care needs, enriching our understanding and informing more tailored interventions.

## Conclusion

The findings of this study revealed that patients reported the highest levels of unmet supportive care needs. Patients encountered more challenges and gaps, particularly in receiving adequate support regarding their physical well-being and health system/information need.

Moreover, the analysis identified several factors as predictors of unmet supportive care needs among the participants. These factors included age, sex, educational status, and stage of cancer. It is recommended to design targeted interventions on improving physical care, enhancing healthcare system accessibility, and providing tailored information based on patient needs. Age- and gender-specific strategies are crucial to ensure the interventions effectively address the unique challenges of various patient groups.

## Data Availability

The dataset supporting the conclusions of this article is included within the article.

## Abbreviations

AOR: Adjusted Odd Ratio
CI: Confidence Interval
COR: Crude Odd Ratio
SD: Standard Deviation
QoL: Quality of life
SCNs': Supportive care needs
TASH: Tikur Anbessa Specialized Hospital
SCNS-SF 24: Supportive care needs: The short-form Supportive Care Needs Survey Questionnaire
